# Targeting CRAC channels in inflammatory bowel disease

**DOI:** 10.15252/emmm.202216489

**Published:** 2022-08-15

**Authors:** Sven Kappel, Christine Peinelt

**Affiliations:** ^1^ Institute of Biochemistry and Molecular Medicine University of Bern Bern Switzerland

**Keywords:** Digestive System, Immunology

## Abstract

Inflammatory bowel disease (IBD) is a collective term for inflammatory diseases of the human gastrointestinal (GI) tract that are characterized by perturbations in the intestinal immune responses. In their study, Letizia *et al* (2022) found an enrichment of CD4^+^ effector T cells, interferon gamma (IFNγ) producing CD8^+^ T cells, regulatory T cells, and innate lymphoid cells (ILC) in the lamina propria (LP) of IBD patients. In these cells, pharmacological inhibition of store‐operated calcium entry (SOCE) reduced cytokine production. In addition, in a murine IBD model, systemic SOCE inhibition reduced IBD severity and weight loss.

## Graphical abstract

S. Kappel and C. Peinelt discuss the study by the laboratories of S. Feske and C. Weidinger (in this issue of EMBO Mol Med) that reports that store‐operated calcium entry controls innate and adaptive immune cell function in inflammatory bowel disease.

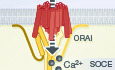

IBD is characterized by inflammation of the digestive tract. While inflammation of the superficial lining of the large intestine is referred to as ulcerative colitis (UC), inflammation in Crohn's disease (CD) can affect the entire GI tract. Symptoms of IBD include diarrhea, unintended weight loss, abdominal pain, and severe internal cramping. In addition, longstanding inflammation due to IBD can lead to the development of colorectal cancer. The incidence of IBD differs among countries but an overall increase in IBD prevalence has been observed over the last years (Kuenzig *et al*, 2022).

While the underlying cause for IBD is still unknown, IBD is characterized by an overshooting immune response and therapies involve anti‐inflammatory drugs, immunosuppressants, biologics, and antibiotics and recent developments aim to boost the physicians therapeutic approaches (Cohen & Rubin, 2021). In IBD, the presence of local immune cells, such as macrophages, dendritic cells (DC), and specific T cell subsets, and cytokine production add to inflammation (Neurath, 2019).

Intracellular calcium ions (Ca^2+^) encode cellular signaling and control many general and immune‐specific functions. Upon receptor stimulation in immune cells, inositol 1,4,5‐trisphosphate (IP_3_) production results in the depletion of intracellular Ca^2+^ stores via intracellular IP_3_ receptors. This drop in Ca^2+^ subsequently leads to cluster formation of stromal interaction molecules (STIM) and the activation of Orai Ca^2+^ channels in the plasma membrane. Besides Orai1 and STIM1, family members Orai2 and Orai3 as well as STIM2 are cell type specifically expressed and form SOCE or the so‐called Ca^2+^ release‐activated Ca^2+^ (CRAC) channels. CRAC channels differ functionally and in their pharmacological profile dependent on their STIM1/STIM2 and Orai1‐3 subunit composition.

SOCE channel activity is important for the innate immune response and controls immune cell signaling in neutrophils, monocytes, macrophages, and mast cells in inflammation and allergy and cytotoxicity of natural killer (NK) cells (Zhou *et al*, 2018; Clemens & Lowell, 2019).

In T cells of the adaptive immune system, SOCE channel signals contribute to activation of different cellular pathways including aerobic glycolysis and mitochondrial and lipid metabolism (Wang *et al*, 2020). SOCE channels control T cell‐mediated immunity and are required for the differentiation and function of T lymphocyte subsets that mediate immunity to infection, inflammation, and cytotoxicity (Zhou *et al*, 2018; Vaeth *et al*, 2020). In a distinct T helper cell subset (T_H_17), SOCE controls expression of genes that are required for mitochondrial function and oxidative phosphorylation thereby regulating pathogenic function of T_H_17 cells (Kaufmann *et al*, 2019). SOCE channel inhibitors have successfully been used to reduce production of pro‐inflammatory cytokines (IFNγ, interleukin IL‐2, IL‐17) by certain T cell subsets from the LP of IBD patients (Di Sabatino *et al*, 2009).

In their recent study, Letizia *et al* (2022) show that per se SOCE is required to induce colitis in a murine model. So far, characterization of immune cell composition within the LP of IBD patients has been limited. Using advanced mass cytometry, the authors could reveal the alterations in immune cell composition of IBD patients. The LP of IBD patients is characterized by elevated numbers of certain T cell subsets including naive T cells, CD4^+^ effector T cells, IFNγ‐producing CD8^+^ T cells, T regulatory cells, and ILC. In addition, in UC patients, prevalence of several B cell subsets was changed.

Inhibition of SOCE by the CRAC channel inhibitor BTP2 dose dependently reduced SOCE and the production of several inflammatory cytokines (e.g., IL‐2, IL‐17 and IFNγ) in CD4^+^ and CD8^+^ effector T cells. SOCE inhibition slightly reduced IL‐6 production of B cells and decreased IFNγ production by myeloid cells. Further analysis revealed that SOCE inhibition by BTP2 resulted in a suppression of tumor necrosis factor α (TNFα) production in most T cell subsets in UC and to a lesser degree in CD. In addition, the production of IFNγ and IL‐2 was reduced by BTP2 treatment in both diseases (Fig 1).

**Figure 1:**
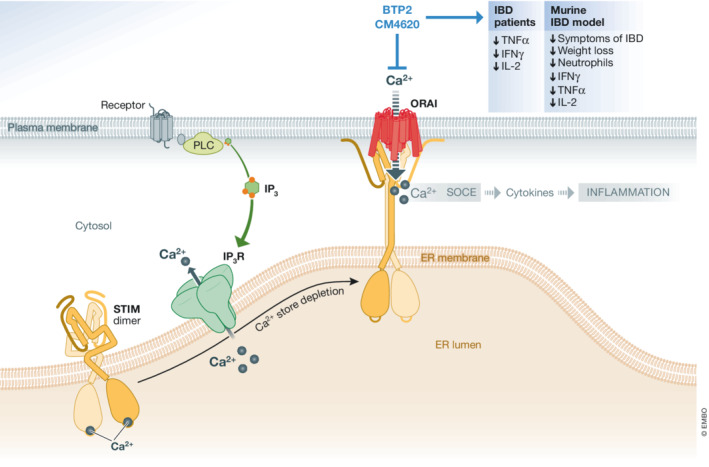
In immune cells, IP_3_ is produced upon receptor stimulation IP_3_ binds to its receptor in the membrane of the endoplasmic reticulum (ER). Ca^2+^ efflux from the ER leads to conformational changes in the stromal interaction molecule (STIM) and cluster formation. In close proximity to the plasma membrane, STIM activates Orai channels that generate a Ca^2+^ signal, the so‐called store‐operated calcium entry (SOCE). Downstream, Ca^2+^ triggers cellular responses, for example, cytokine production. Pharmacological inhibition of SOCE by BTP2 or CM4620 reduces production of inflammatory cytokines in humans and mice, and reduces symptoms of inflammatory bowel disease (IBD) in a murine model.

Both, T cells and non‐T cells (i.e., NK cells, ILC, and B cells) require functional SOCE signaling. SOCE inhibition reduced expression of IL‐6 by B cells, expression of TNFα by ILC and NK cells, and expression of IFNγ by myeloid and NK cells from IBD patients. In conclusion, the authors demonstrate that SOCE in these non‐T cells contributes to the inflammatory milieu in the LP of IBD patients.

To test whether BTP2 alters the function of intestinal epithelial cells, Letizia *et al* (2022) tested BTP on murine and human colonic organoids and 2D monolayers and found no impairment of viability, differentiation, and barrier function of the epithelial cells. These findings suggest that CRAC channel targeting in IBD specifically reduces inflammation without affecting healthy epithelial cells.

The authors tested another specific CRAC channel inhibitor, CM4620 that is currently in a clinical trial phase I for COVID‐19‐associated pulmonary inflammation and clinical trial phase I/II for acute pancreatitis. CM4620 was found to be even more potent in reducing inflammatory cytokine production than BTP2.

In addition, systemic application of CM4620 was effective in reducing inflammation in a murine IBD model demonstrating the potential of targeting CRAC channel function as therapeutic approach against IBD.

Taken together, this joined study by the labs of Stefan Feske and Carl Weidinger highlights the specificity of inflammatory cytokine reduction by SOCE inhibition in IBD‐associated cells and opens up a novel treatment strategy in IBD.
